# Impact of ‘Stretch’ Targets for Cardiovascular Disease Management within a Local Pay-for-Performance Programme

**DOI:** 10.1371/journal.pone.0119185

**Published:** 2015-03-26

**Authors:** Utz J. Pape, Kit Huckvale, Josip Car, Azeem Majeed, Christopher Millett

**Affiliations:** Department of Primary Care and Public Health, Imperial College London, London, United Kingdom; Azienda Ospedaliero-Universitaria Careggi, ITALY

## Abstract

Pay-for-performance programs are often aimed to improve the management of chronic diseases. We evaluate the impact of a local pay for performance programme (QOF+), which rewarded financially more ambitious quality targets (‘stretch targets’) than those used nationally in the Quality and Outcomes Framework (QOF). We focus on targets for intermediate outcomes in patients with cardiovascular disease and diabetes. A difference-in-difference approach is used to compare practice level achievements before and after the introduction of the local pay for performance program. In addition, we analysed patient-level data on exception reporting and intermediate outcomes utilizing an interrupted time series analysis. The local pay for performance program led to significantly higher target achievements (hypertension: p-value <0.001, coronary heart disease: p-values <0.001, diabetes: p-values <0.061, stroke: p-values <0.003). However, the increase was driven by higher rates of exception reporting (hypertension: p-value <0.001, coronary heart disease: p-values <0.03, diabetes: p-values <0.05) in patients with all conditions except for stroke. Exception reporting allows practitioners to exclude patients from target calculations if certain criteria are met, e.g. informed dissent of the patient for treatment. There were no statistically significant improvements in mean blood pressure, cholesterol or HbA1c levels. Thus, achievement of higher payment thresholds in the local pay for performance scheme was mainly attributed to increased exception reporting by practices with no discernable improvements in overall clinical quality. Hence, active monitoring of exception reporting should be considered when setting more ambitious quality targets. More generally, the study suggests a trade-off between additional incentive for better care and monitoring costs.

## Introduction

Pay for performance programmes are being adopted in a growing number of countries as a quality improvement tool [1,2]. In 2004, the United Kingdom introduced the Quality and Outcomes Framework (QOF) which primarily aimed to improve the management of common chronic conditions, such as diabetes and stroke, in primary care [3]. Studies suggest that QOF was associated with modest improvements in quality of care [4–7], although gains are not evident in all incentivised clinical areas and adverse effects have been seen in specific subpopulations like older patients or patients in deprived areas [8–11]. Exception reporting, a mechanism for practitioners to temporarily exclude patients for whom targets are clinically inappropriate, further complicates assessment of the impact of QOF [12].

Proposals to set aside part of the national QOF budget to develop local pay for performance programmes have not been implemented [13]. Potential advantages of local programmes include the ability to target local health needs, reduce health inequalities and foster greater clinical engagement for quality improvement [14]. The largest local programme is QOF+, which was launched in the London borough of Hammersmith and Fulham in September 2008 (see text box for description in the reference [14]). A key objective of the programme is to accelerate improvements in existing national QOF targets by setting more ambitious local payment thresholds (‘stretch targets’) for achieving specific intermediate outcomes for diabetes, hypertension, coronary heart disease (CHD) and stroke.

This study evaluates the impact of QOF+ stretch targets on intermediate outcomes in patients with cardiovascular disease and diabetes. As part of this, we assess whether setting more ambitious targets led to increased exclusion (‘exception reporting’) of patients from the pay for performance programme.

## Methods

### Setting

QOF+ was launched by Hammersmith and Fulham primary care trust in West London during September 2008. The primary care trust serves around 180 000 residents covered by 31 general practices and has two main acute hospitals. The resident population is young (one third aged 20–34 years), mobile (12% turnover a year), and culturally diverse (22% from ethnic minorities) with considerable income inequality.

### Data

Annual patient-level data on all adult patients (≥ 18 years) registered at 31 Hammersmith and Fulham general practices during financial years 2004/05 to 2010/11 were extracted from electronic medical records. The de-anonymized and de-identified extract includes anonymised information on patient demographics, clinical diagnoses and clinical measurements [15]. As the patient-level data does not contain identifiers or patient sensitive information, individual patient consent was not required. Publicly available annual practice-level data of QOF performance for all practices in England for the years 2006/07 to 2010/11 was obtained from the NHS Information Centre. The dataset does not contain patient-level data. Ethics approval was granted by London Queen Square Research Ethics Committee. As QOF+ was introduced in December 2008, we dropped data for the 2008/09 financial year from both the patient and practice level datasets.

### Study variables

Our main outcome measures were mean values and achievement of clinical targets for blood pressure, total cholesterol and HbA1c. For multiple measurements within a period, the last measurement was used for compatibility with the calculation of the performance indicators. Covariates in our patient level analyses included age, gender, ethnicity, body mass index, number of cardiovascular comorbidities and area socio-economic status (based on the index of multiple deprivation 2007) [16]. Age was divided into three categories: 18 to 44, 45 to 64 and 65+ years. Body mass index was split into the three categories below 25, between 25 and 30 and above 30 kg/m^2^. Ethnicity is categorized into White, Black (Caribbean and African), South Asian (Indian, Pakistani and Bangladeshi) and Other (including Chinese). Based on indicators for coronary heart disease, diabetes, hypertension, stroke or transient ischemic attack, atrial fibrillation and heart failure, we calculated the number of cardiovascular comorbidities (0,1,2+) per patient. Annual data on whether a patient had been exception reported from QOF/QOF+ was obtained.

Patient records with diastolic blood pressure not inside the interval 20 and 160 (excluding the limits) were discarded. Similarly, records with systolic blood pressure not inside the interval 30 to 250 (excluding the limits) were discarded. Records with cholesterol values greater or equal to 15 were removed as well as HBA1c values greater or equal to 20. Body mass index has an additional category for missing values, indices below 10 and above 70 due to the high number of records with missing values. Note that missing values cannot be imputed by interpolation because all patients with at least one missing value for body mass index have no body mass index given in any year.

As there were changes in the business rules for QOF over the study period we applied the same version (version 16 rule set) across all years.

### Statistical Analyses

We conducted three different analyses at an a-priori chosen significance level of 5%. The first analysis tested whether QOF+ was associated with improvements in target achievement relative to national trends. The second analysis tested whether QOF+ was associated with an increase in exception reporting and whether any changes in exception reporting influenced target achievement. The third analysis investigated the impact of QOF+ on actual clinical values.

#### Analysis 1: National Comparison

The national comparison is performed using practice-level data. The outcome measure is the percentage of patients reaching QOF targets. The treatment group contains Hammersmith and Fulham practices while the comparison group consists of all remaining practices in England. With the intervention of QOF+ in 2009, we compare the performance of treatment and comparison practices before and after the intervention. This involves a difference-in-difference approach, which is a quasi-experimental methodology, to isolate the intervention effect by controlling for secular trends and changes affecting both groups. Few practices (between 677 (8%) and 1103 (13%) of 8641 practices—depending on the indicator), which do not have data for all years, are removed from the dataset to make the analysis robust against mixing effects. As patients’ registration with practices is not random, we use a mixed effect model with random effects for practices. We model residuals with a 1-year lag correlation structure to allow for autocorrelation. The model is applied separately to different QOF+ targets. As an additional comparison, we also run the analysis for indicators that were not incentivised under QOF+ (COPD8, COPD10, HF2, HF3; see Table 1 for a definition of included indicators). A difference-in-difference approach assumes that the pre-intervention trends of treatment and control group are the same. We verify the parallel assumption by testing for a significant difference in the pre-intervention time points between treatment and control group. Similar to the difference-in-difference model, we employ the same mixed effect model but cannot control for auto-correlation with only two time points.

**Table 1:** Definition of indicators.

**BP5**	Percentage of patients with hypertension with a measured blood pressure of less or equal to 150/90 for the last measurement since 9 months.
**CHD6**	Percentage of patients with coronary heart disease with a measured blood pressure of less or equal to 150/90 for the last measurement within 15 months.
**CHD8**	Percentage of patients with coronary heart disease with a measured total cholesterol of less or equal to 5mmol/l for the last measurement within 15 months.
**CHD10**	Percentage of patients with coronary heart disease who are currently treated with a beta blocker (unless contraindication).
**COPD8**	Percentage of patients, who had an influenza immunisation in the preceding 1 September to 31 March.
**COPD10**	Percentage of patients with a record of FeV1 in the previous 15 months.
**DM12**	Percentage of patients with diabetes with a measured blood pressure of less or equal to 145/85 for the last measurement within 15 months.
**DM17**	Percentage of patients with diabetes with a measured total cholesterol of less or equal to 5mmol/l for the last measurement within 15 months.
**DM24**	Percentage of patients with diabetes with a measured HbA1c of less or equal to 8% for the last measurement within 15 months.
**DM25**	Percentage of patients with diabetes with a measured HbA1c of less or equal to 9% for the last measurement within 15 months.
**HF2**	Percentage of patients with a heart failure confirmed by an echocardiogram or specialist assessment.
**HF3**	Percentage of patients, who are currently treated with ACE inhibitor or Angiotensin Receptor Blocker without any contra-indications.
**STROKE6**	Percentage of patients with a history of stroke or TIA with a measured blood pressure of less or equal to 150/90 for the last measurement within 15 months.
**STROKE8**	Percentage of patients with a history of stroke or TIA with a measured total cholesterol of less or equal to 5mml/l for the last measurement within 15 months.

#### Analysis 2: Exception Reporting and Changes in Target Achievement

To understand the extent that exception reporting may have accounted for improvements in achievement of QOF+ targets, we test for significant changes in the number of exception reported patients. Without a control group available, we identify changes by comparing the fraction of exception reported patients relative to all patients before and after the introduction of QOF+. The fraction aggregated on the practice level serves as dependent variable. Under the assumption that exception reported patients are usually not controlled, a significant change in the fraction of exception reported patients upon introduction of QOF+ could explain results from the first analysis. Similarly, we test for changes in the fraction of controlled patients relative to all non-exception reported patients. An interrupted-time-series analysis is used to control for a secular trend in both analyses. The estimated fixed effect model allows clustered standard errors on the practice level. As we cannot control for patient characteristics at the practice-level, we restrict the sample to patients who have measurements in all years. Therefore, the patient characteristics do not change over time making the estimator invariant to patient inflows or outflows.

#### Analysis 3: Clinical Outcomes

This analysis is constructed to capture the impact of QOF+ on the clinical measurements that are the subjects of the included indicators. The impact of QOF+ is measured as an additive effect in the years 2010 and 2011. The change is estimated relative to the 2-year pre-QOF+ period from 2007 to 2008. This time restriction is motivated by the observation that QOF years 2004 to 2008 do not follow a linear secular trend but are subject to trend changes in 2006 and 2007 for clinical outcomes but not for the rate of exception reporting.

We accommodate the multi-level nature of the data by employing a hierarchical mixed effect model estimated with a restricted maximum likelihood approach. Within-patient measurements are correlated and modelled by a patient random effect. Clustering of patients within practices is captured by a practice random effect. We adjust the correlation structure of the residuals in the mixed effect model by allowing for one year lagged autocorrelation. The remaining covariates discussed above are included as fixed effects. The analysis is conducted for three groups of patients. For the population-level effect, all patients are included. The differential effect on exception reported patients is estimated from the corresponding subset of exception reported patients. The third group consists of the non-exception reported patients. The analysis is conducted separately for different groups and indicators.

## Results

The patient characteristics for the 31 practices in Fulham and Hammersmith are described in Table 2. The gender of patients was well balanced except for coronary heart disease with 64% male patients. Most patients are from white ethnic backgrounds followed by black and South Asian ethnicities. The mean number of cardiovascular comorbidities was approximately two with diabetes patients having the highest number of comorbidities (2.2) and stroke patients the least number of comorbidities (1.7). Mean BMI ranged from 28.0 kg/m^2^ for stroke patients to 29.5 kg/m^2^ for diabetes patients.

**Table 2:** Number of patients per disease groups and their average age, proportion of females, ethnicity, number of cardiovascular disease co-morbidities and body-mass-index for 2011.

	Diabetes	Hypertension	CHD	Stroke
**N**	6142	16834	3176	1630
**Age (mean)**	61.7	64.9	70.6	71.2
**% female**	46%	52%	36%	52%
**Ethnicity**				
**White (%)**	45%	58%	64%	67%
**Black (%)**	23%	18%	9%	13%
**South Asian (%)**	17%	10%	14%	7%
**Number of CVD CM**	2.2	2.0	1.9	1.7
**BMI (mean)**	29.5	28.9	28.3	28.0

### Analysis 1: National Comparison

**Fig 1:**
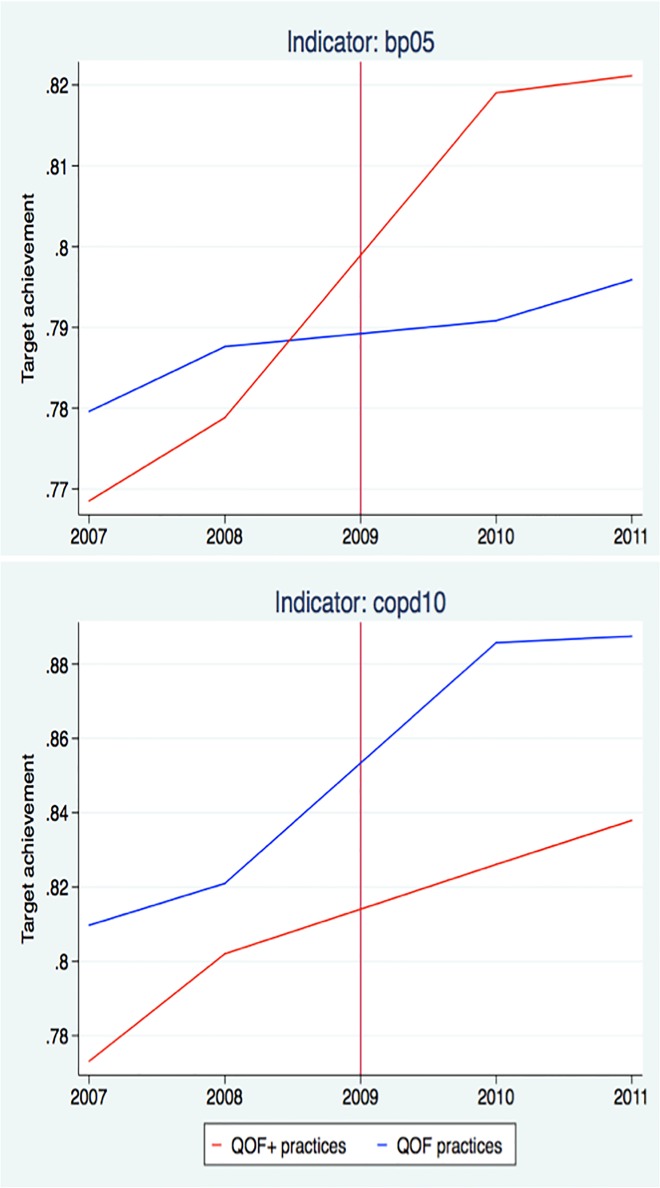
Target achievement rates for a stretched QOF+ indicator BP5 and a QOF indicator COPD10 for QOF+ and QOF practices.

Table 3 shows the results of the difference-in-difference approach. In the first column, we observe that all but four indicators (CHD8, DM17, STROKE8 and COPD8) treatment and control practices have a similar pre-intervention trend as required by a difference-in-difference approach based on a conservative significance level of 10%. Fig. 1 visualizes the trend for BP5 (incentivised within QOF+) and COPD10 (not incentivised within QOF+). The effect of interest is the differential impact of QOF+ on treatment practices in the second column of Table 3. All QOF+ stretched indicators have a significant and positive coefficient. For example, BP5 target achievement rates increase by additional 3.7% points for QOF+ practices (p-value <0.001). At the same time, the four control indicators for COPD and CHF, which are not subject to additional incentives in QOF+, do not show significant differential effects between QOF+ and control practices. As an example, COPD10 target achievement rates do not differ significantly between QOF+ and control practices (p-value 0.176). Thus, for all stretched indicators, which follow a parallel pre-intervention trend, the target achievement rates significantly increased upon introduction of QOF+; while target achievement rates of unmodified indicators were not affected.

**Table 3:** Results for the national comparison based on a difference-in-difference model.

Indicator		Parallel Assumption	QOF+ Effect
**BP5**	Effect	-0.010	0.037
	p-value	0.402	<0.001
**CHD6**	Effect	0.000	0.029
	p-value	0.992	<0.001
**CHD8**	Effect	-0.052	0.080
	p-value	<0.001	<0.001
**DM12**	Effect	0.006	0.024
	p-value	0.701	0.061
**DM17**	Effect	-0.050	0.061
	p-value	<0.001	<0.001
**STROKE6**	Effect	0.001	0.032
	p-value	0.951	0.002
**STROKE8**	Effect	-0.044	0.096
	p-value	0.009	<0.001
**COPD8**	Effect	-0.016	-0.002
	p-value	0.083	0.842
**COPD10**	Effect	-0.028	-0.028
	p-value	0.203	0.176
**HF2**	Effect	0.011	-0.021
	p-value	0.470	0.198
**HF3**	Effect	0.002	-0.015
	p-value	0.872	0.305

The rows contain different indicators with effect size and p-value. The parallel assumption is tested in the first column. The QOF+ effect is calculated as the interaction effect of treatment and intervention isolating the impact of QOF+ shown in the last column.

### Analysis 2: Exception Reporting and Indicator Change

Changes in target achievement can either be caused by changes in the number of exception reported patients or by an increase in the number of well-controlled patients. We can disentangle these effects by analysing changes in the number of exception reported patients and controlled patients. First, we estimate the changes in the proportion of exception reported patients relative to all patients. Table 4 shows the baseline from 2005 and the secular annual change of exception reported patients in the first two columns. Only the indicator BP5 has a significant change in exception reporting over time, decreasing by 1.3% points (17% relative to the baseline 2005) per year (p-value <0.001). This decreases the rate of exception reporting for BP5 from over 8% in 2005 to 4% in 2008 before the introduction of QOF+.

**Table 4:** Change in the fraction of exception reported and controlled patients relative to all patients in the corresponding disease group upon introduction of QOF+.

		Exception Reporting			Controlled Patients		
Indicator		Base-line	Secular Trend	QOF+ Baseline	Base-line	Secular Trend	QOF+ Baseline
**BP5**	Effect	0.080	-0.013	0.053	0.765	0.019	0.006
	p-value		<0.001	<0.001		0.002	0.859
**CHD6**	Effect	0.024	-0.003	0.024	0.837	0.011	0.037
	p-value		0.143	0.028		0.016	0.278
**CHD8**	Effect	0.057	-0.003	0.037	0.723	0.026	0.016
	p-value		0.438	0.029		0.001	0.689
**DM12**	Effect	0.039	-0.001	0.025	0.719	0.021	0.026
	p-value		0.856	0.090		0.021	0.564
**DM17**	Effect	0.060	-0.001	0.024	0.722	0.035	-0.018
	p-value		0.884	0.258		<0.001	0.666
**DM24**	Effect	0.085	0.001	0.060	0.725	0.015	0.002
	p-value		0.910	0.018		0.005	0.968
**DM25**	Effect	0.062	0.001	0.043	0.822	0.015	0.003
	p-value		0.891	0.049		0.002	0.934
**STROKE6**	Effect	0.046	-0.005	0.034	0.836	0.003	0.097
	p-value		0.335	0.242		0.693	0.040
**STROKE8**	Effect	0.076	0.001	0.017	0.644	0.042	-0.007
	p-value		0.858	0.651		0.001	0.897

The rows contain different indicators with the baseline in the first column, the secular trend effect in the second column and the change with the introduction of QOF+ in the third column.

With the introduction of QOF+, the fraction of exception reported patients increases significantly for five indicators (third column in Table 4); BP5 (5.3% points, p-value <0.001), CHD6 (2.4% points, p-value 0.028), CHD8 (3.7% points, p-value 0.029), DM24 (6% points, p-value 0.018) and DM25 (4.3% points, p-value 0.049) between 2 and 6 percentage points. The QOF+ effect represents the average change from before 2009 to after 2009 with consideration of the secular trend. For example, BP5 would have further decreased by 1.3% points per year without the introduction of QOF+. However, the introduction of QOF+ increased the rate of exception reporting in average for 2010 and 2011 by 5.3% points relative to the expected secular decrease.

Table 4 shows the impact of QOF+ on the proportion of controlled patients relative to all non-exception reported patients in the last three columns. All indicators but STROKE6 show a significant secular improvement in the proportion of controlled patients ranging from 1.1% points (CHD6, p-value 0.016) to 4.2% points (STROKE8, p-value 0.001). For these indicators, there is no additional significant change of the proportion of controlled patients upon the introduction of QOF+ (p-values ≥ 0.278). Only STROKE6 has no significant secular trend (p-value 0.693) but the proportion of controlled patients significantly improved upon introduction of QOF+ by an average of 9.7% points (p-value 0.04) in 2010 and 2011.

### Analysis 3: Clinical Outcomes

**Fig 2:**
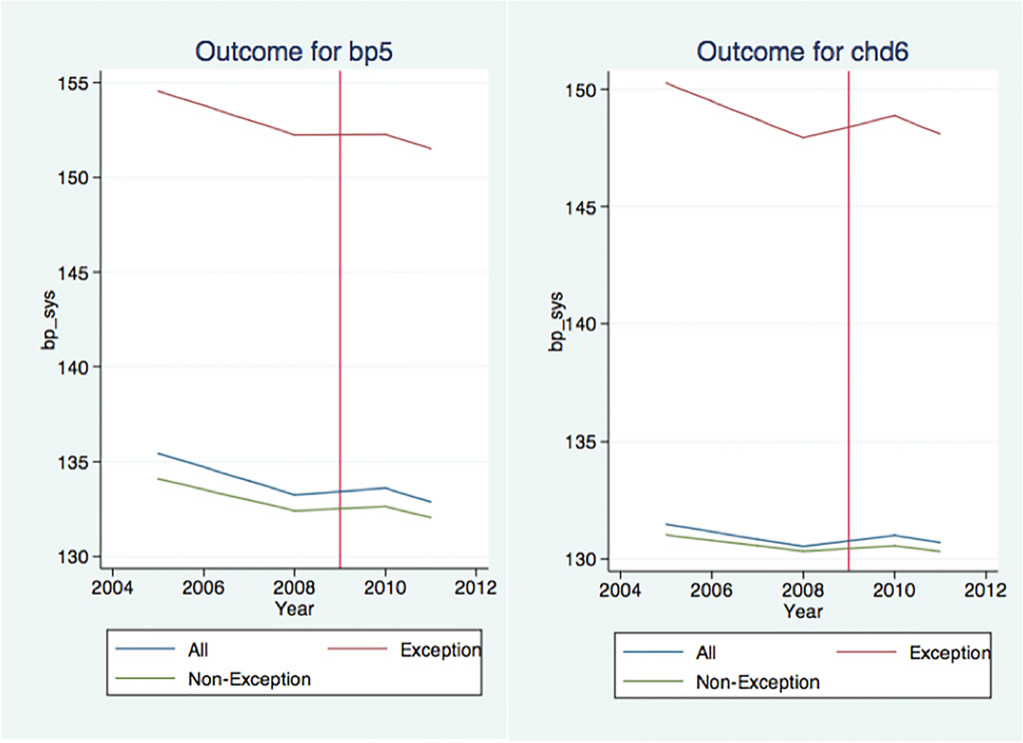
Systolic blood pressure as clinical outcome for hypertension patients (left) and coronary heart disease patients (right). The panels compare the blood pressure between all patients, exception reported patients and non-exception reported patients.

The impact of QOF+ on clinical outcomes is shown in Table 5 subdivided for different groups of patients with an illustration in Fig. 2. The introduction of QOF+ was associated with a statistically significant increase in blood pressure in patients with hypertension (diastolic 0.54 mm Hg, p-value <0.001; systolic 1.81mm Hg, p-value <0.001) and diabetes (diastolic 0.80mm Hg, p-value 0.002; systolic 1.70mm Hg, p-value <0.001) and an increase in systolic blood pressure in patients with stroke (1.90mm Hg, p-value 0.026). For exception reported patients, only the diastolic blood pressure worsens significantly for stroke patients (6.92mm Hg, p-value 0.005). For non-exception reported patients, the blood pressure deteriorates significantly for diabetes patients (diastolic 0.64mm Hg, p-value 0.017; systolic 1.50mm Hg, p-value 0.001) as well as the systolic blood pressure for hypertension patients (1.34mm Hg, p-value <0.001). However, the cholesterol value improves significantly for coronary heart disease (-0.07mmol/l, p-value 0.013).

**Table 5:** Change in the bio-parameter for the indicator shown in the row for the group of patients mentioned in the column (all patients, exception reported patients, non-exception reported patients).

		All Patients		Exception Reported		Non-Exception Reported	
Disease	Outcome	Secular Trend	QOF+ Change	Secular Trend	QOF+ Change	Secular Trend	QOF+ Change
**Hyper-tension**	Diastolic BP	-0.509	0.541	-0.781	1.324	-0.400	0.239
	p-value	<0.001	<0.001	<0.001	0.054	<0.001	0.120
	Systolic BP	-0.731	1.813	-0.770	1.566	-0.561	1.335
	p-value	<0.001	<0.001	0.005	0.162	<0.001	<0.001
**CHD**	Diastolic BP	-0.328	0.201	-0.127	0.342	-0.284	-0.053
	p-value	<0.001	0.565	0.787	0.855	0.001	0.881
	Systolic BP	-0.314	1.113	-0.778	2.497	-0.235	0.699
	p-value	0.031	0.054	0.401	0.495	0.101	0.217
	Cholesterol	-0.066	-0.032	-0.081	0.050	-0.058	-0.074
	p-value	<0.001	0.279	0.004	0.644	<0.001	0.013
**Diabetes**	Diastolic BP	-0.332	0.804	-0.192	-0.193	-0.298	0.641
	p-value	<0.001	0.002	0.574	0.883	<0.001	0.017
	Systolic BP	-0.282	1.696	0.286	-1.524	-0.260	1.503
	p-value	0.014	<0.001	0.637	0.509	0.023	0.001
	Cholesterol	-0.088	0.000	-0.063	-0.045	-0.083	-0.028
	p-value	<0.001	0.992	0.034	0.692	<0.001	0.273
	HbA1c	-0.042	-0.001	-0.034	-0.152	-0.042	-0.026
	p-value	<0.001	0.969	0.464	0.382	<0.001	0.491
**Stroke**	Diastolic BP	-0.514	0.338	-2.318	6.918	-0.417	0.007
	p-value	<0.001	0.502	<0.001	0.005	0.001	0.989
	Systolic BP	-0.939	1.901	-2.018	7.757	-0.868	1.618
	p-value	<0.001	0.026	0.051	0.057	<0.001	0.053
	Cholesterol	-0.092	-0.006	-0.028	-0.101	-0.090	-0.037
	p-value	<0.001	0.896	0.388	0.415	<0.001	0.414

The secular 2-year pre-QOF+ trend effect is shown in the first group column and the change of the bio-parameter upon introduction of QOF+ in the second group column.

## Discussion

### Main findings

The introduction of local pay for performance programme (QOF+) had a significant impact on target achievement for quality indicators subject to enhanced financial incentives. Most of the improvements in target achievement were due to increases in exception reporting. The programme was not associated with discernable improvements in overall clinical quality.

### How this fits with previous research

The effect of setting more demanding targets within existing pay for performance programmes on clinical performance has been little investigated. One previous study examined the impact of an increase in the upper payment threshold for influenza immunization in CHD patients from 85% to 90% in QOF during 2006/07 [17]. The findings suggest that this change was associated with modest increases in the proportion of CHD patients immunised (0.41%, CI: 0.25–0.56%) but that the proportion exception reported (0.26%, CI: 0.12–0.40%) for this indicator also increased. Our study builds on this previous study by demonstrating that a local QOF+ programme, which set more ambitious payment targets, was associated with increased exception reporting across different clinical outcomes and more disease groups.

### Strengths and Limitations

Our study benefits from several strengths in the design of the analysis. Unlike other pay for performance studies, our models take the underlying secular trends into account using a time-series approach [18]. In addition, we adjust for important covariates. In contrast to an evaluation of the national QOF program, we focus on a local pay for performance scheme, which allows us to compare indicators with non-intervention sites using the more robust difference-in-difference approach. In addition, the increased rate of exception reporting cannot be explained by a change of patient composition since the analysis was conducted for patients registered in all years.

Except for the results of the difference-in-difference approach (Analysis 1), our findings may be influenced by other reforms occurring at the same time of QOF+. However, we are not aware of any major quality improvement programmes introduced for CHD, hypertension or stroke care at the time that QOF+ was implemented.

In the analysis of clinical outcomes, we observed that at a population level, QOF+ may have had limited or even negative impacts on risk factor control. This reflects findings from national and local data which suggest that secular improvements in clinical outcomes before the introduction of QOF+ had started to stagnate [5,7]. Without a control group for clinical outcomes not affected by QOF+ (data unavailable), we cannot disentangle a secular stagnation effect from the impact of QOF+. On the other hand, the deterioration can be due to neglect of patients who are exception reported or other subgroups.

### Policy implications

Improvements in target achievement associated with the introduction of a local pay for performance programme with more ambitious payment targets than those set in national QOF were mainly attributed to increased exception reporting by practices. Exception-reported patients are less likely to achieve clinical targets [19] and the impact of their exclusion is to increase the cost to the scheme of each patient who does meet the targets [20]. Therefore, implementation of pay-for-performance programmes should be accompanied by measures to prevent higher exception reporting. This requires defining an appropriate level of exception reporting, which is notoriously difficult to assess; or an active monitoring program, which contributes to the overhead costs. More generally, the study suggests a trade-off between additional incentives for better care and monitoring costs, which should be considered already in the design of the program.

## Supporting Information

S1 Dataset Supporting information for article. (DTA)
